# The Attention Modulation on Timing: An Event-Related Potential Study

**DOI:** 10.1371/journal.pone.0066190

**Published:** 2013-06-24

**Authors:** Yunzhe Liu, Dandan Zhang, Jing Ma, Dan Li, Huazhan Yin, Yuejia Luo

**Affiliations:** 1 Institute of Affective and Social Neuroscience, Shenzhen University, Shenzhen, China; 2 Faculty of Psychology, Southwest University, Chongqing, China; 3 Department of Psychology, Chong Qing Normal University, Chongqing, China; Cardiff University, United Kingdom

## Abstract

The present study examined the neural mechanisms of attention modulation on timing using ERP and sLORETA measurements in a dual-task paradigm. We parametrically varied the attention to the durations of a 1000-Hz pure tone and further localized the cortical regions that were sensitive to the attention modulation on timing. Results demonstrated that the attention modulation might happen at early stage, approximately 200 ms after stimulus presentation. The P2 component at frontal area served as an early neural correlate of attention effects on timing. More importantly, the contingent negative variation (CNV) appeared at fronto-central area was sensitive to the attention effect. In addition, the supplementary motor area (SMA) was assumed to be one of the key regions for selectively attending to and estimating time. These findings provide temporal and spatial correlates of attention-modulated time processing and potentially help to investigate the neural mechanisms of patients with time perception deficits.

## Introduction

Attention modulation on timing is an important issue in time perception [1]. According to the timing model based on pulse accumulation [2], attention effects on timing refer to the phenomenon that the more attention is paid to time, the more pulses are accumulated [3,4]. Empirically, when one person allocates more attention to the target duration, he/she judges this duration with a longer duration [4]; any attention shift from the target duration results in shorter estimates [5].

Although the pulse-accumulation model of timing can accurately describe a large part of timing phenomena in cognitive psychology, it is considered no more than a good metaphor of actual time perception mechanism [6]. Beyond this experience theory model, recently, more researchers focused on the neural mechanisms of time perception [6]. Using functional magnetic resonance imaging (fMRI) technique, Coull et al. [7] showed that the blood-oxygen-level-dependent (BOLD) signal in supplementary motor area (SMA) positively correlated with the amount of attention paid to timing. Also, in a time reproduction task, where the duration of one target was first coded and then retrieved, the SMA was the only area involved in both stages and thus was considered as the structure responsible for the accumulation process [8]. Regarding the experimental evidences collected using event-related potentials (ERPs), the contingent negative variation (CNV) is a correlate of brain electrical activity associated with a predictable time interval between two events of interest [9]. The CNV amplitudes are larger when subjects pay attention to the duration versus other properties of the targets [10,11]. Macar and Vidal [10,12] considered the CNV as a neural signature of the temporal accumulator, which performs based on the number of activated neuronal units and is influenced by the amount of attention attributed to timing. However, as reviewed by van Rijn and colleagues [13], a direct link between CNV and the temporal accumulator could not be straightforwardly interpreted. Some studies thus alternatively suggested that the enhanced CNV amplitudes might reflect decision processes involved in interval timing [14–18]. The exact nature of the relation between CNV and the underlying timing mechanisms is still a topic of discussion. The current research did not aim to decide between the decision-making hypothesis and the time estimation hypothesis. Instead, our main concern was the attention effect on timing, i.e., pulse-accumulation phase. Besides the CNV component, researchers also found that an early positive component, at approximately 200 ms post-stimuli, was relevant to attention modulation on timing [19–23]. This P2 component usually shows larger amplitudes when more attention is allocated to time duration rather than other properties of auditory stimuli [20,21,23].

In most of the previous studies associated with the attention modulation on time perception, the differences of physical features of target stimuli (e.g., visual intensity [19] and auditory pitch [20]) might contaminate attention effects on timing. The current study examined the ERPs evoked by two pure tones with different durations but in the same pitch (i.e., 1000 Hz), thus the potential confounding influences due to the physical features beyond time duration can be excluded. Furthermore, most studies investigated attention modulation on timing under two attention conditions, i.e., “with attention to” vs. “with no attention to” the targets [20–22]. In our opinion, the sensitivity of the attention modulation effects can be more comprehensively demonstrated by showing that gradual variation of the attention amount to targets produces a corresponding gradual change in brain activity [7]. In the current study, we parametrically varied the attention amount (in five levels) to the duration vs. the pitch of a pure tone in a dual-task paradigm. The time course and the neural sources of the P2 and CNV components were explored in response to the two tones with the same pitch. We hypothesized that a linear increase in attention to timing would be accompanied by a corresponding increase in the amplitudes of CNV and/or P2, and that the ERP components sensitive to attention effects on timing may have a neural source located in (or near) SMA [11,24].

## Method

### Participants

Fifteen healthy undergraduates (seven males) aged 18-23 years (mean age = 21.2) from Southwest University (Chongqing, China) were recruited as paid participants of the study. All participants had normal or corrected to normal vision and audition, and all were right-handed according to Oldfield criteria [25]. No participant had a history of physiological or psychological disorder. This study had been approved by the IRB at Southwest University. All subjects gave written informed consent in our experiment.

### Procedure

#### *Practice phase Ⅰ*

**Figure 1:**
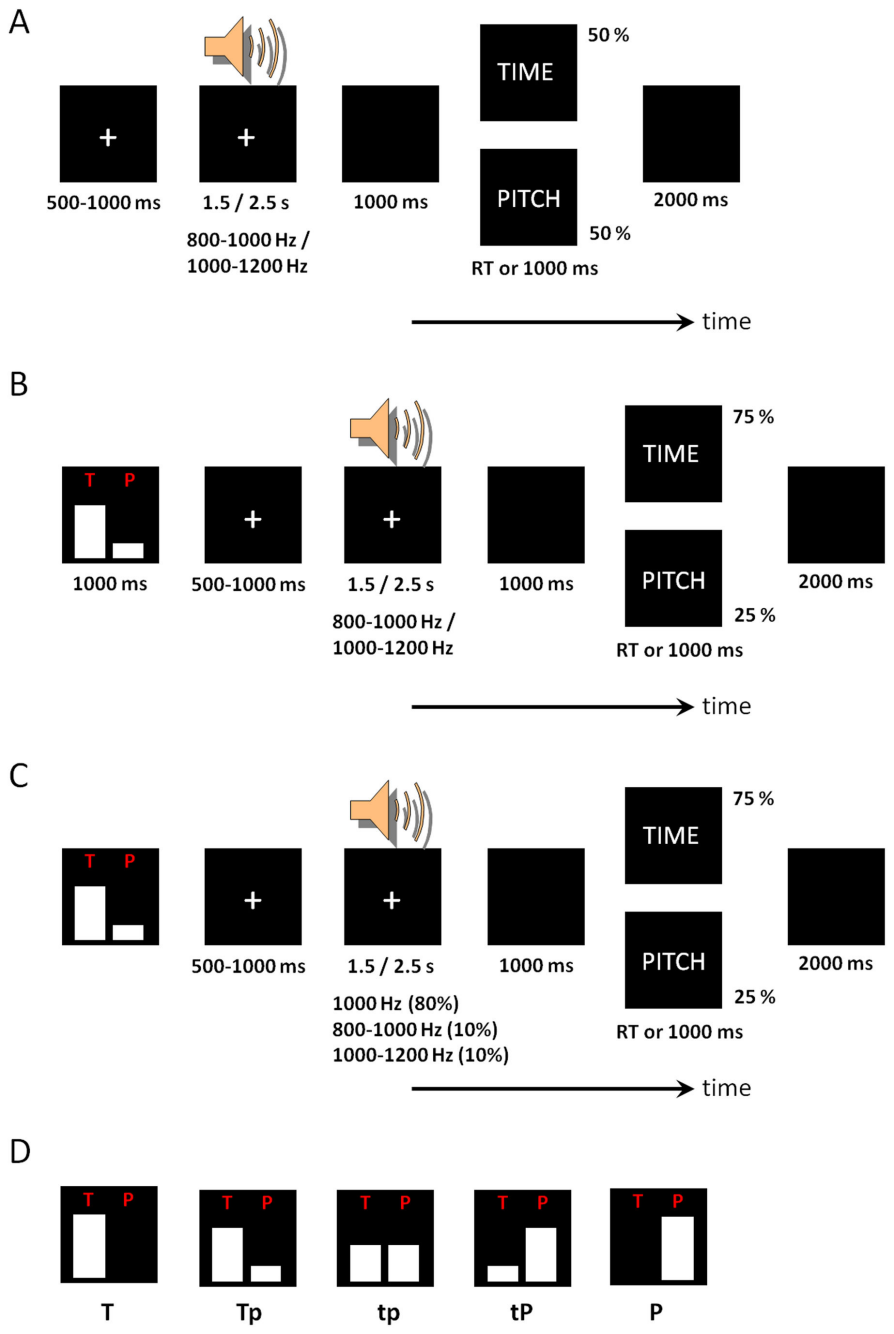
Illustration of the experimental procedure. A, Practice phase I. B, Practice phase ii. C, the formal experiment. The ERP data were collected in response to the 1000-Hz tones with 1.5- and 2.5-s durations. D, the five attentional cues used in this study.

This phase aimed at memory consolidation of the target duration and the pitch of the stimuli (Figure 1A). Two standard auditory stimuli (1000-Hz tones with 1500 ms and 2500 ms durations) were successively presented in five pairs. Then, the tone was presented with varied durations (1500 or 2500 ms) or pitches (800-1000 Hz or 1000-1200 Hz) with equal probability. Participants were instructed to make a response to the stimulus duration (short or long? i.e., time discrimination) or the stimulus pitch (identical with the standard one? i.e., pitch discrimination). Responses were given by the index and middle fingers of the right hand (index finger = shorter/lower; middle finger = longer/higher). This practice phase was repeated until the accurate rate of 80% was reached.

#### *Practice phase Ⅱ*

This phase aimed to familiarize the participants with the experimental task (Figure 1B). There were five attention conditions/blocks in this study. At the beginning of each attention block, the participants were instructed to allocate different amounts of attention to the time duration and pitch of the auditory stimuli. As shown in Figure 1D, the attention cue directed the subjects to attend to the stimulus time (T condition), to the time more than the pitch (Tp condition), to both features equally (tp condition), to pitch more than time (tP condition), or to pitch only (P condition). Participants answered the same questions (time or pitch discrimination in one trial) as in practice phase I. Five attention blocks were included in the practice, each containing 20 trials. The probability of the questions (TIME or PITCH corresponded to the type of attentional cue in each block. This practice phase was repeated until the accurate rate of 80% was reached.

#### *Formal experiment*

The experiment procedure was similar to that in Practice phase II, but with one key exception: there were 80% trials presenting standard 1000-Hz pitch, and 10% trials presenting lower (800-1000 Hz) and higher (1000-1200 Hz) pitches, respectively (Figure 1C). No feedback was provided during the formal experiment [10]. Responses with latencies less than 1000 ms were considered valid, since slow responses may not reflect the participants’ spontaneous feelings [10]. The order of blocks was counterbalanced across participants in Latin square design. Each block contained 80 trials. Thus there were totally 320 valid trials (80%) in the five attention conditions (excluding the 80 deviant trials (20%) with stimulus pitches lower or higher than 1000 Hz). The two standard stimuli were presented three times at the beginning of each block to prevent the participants from forgetting them. Blocks were separated by self-terminated breaks.

### EEG recording and analysis

Brain electrical activity was recorded from 64 scalp sites using tin electrodes mounted in an elastic cap (Brain Product), with the average reference electrodes on the left and right mastoids and a ground electrode on the medial frontal region. Electrooculographic (EOG) data were recorded supra- and infra-orbitally (vertical EOG), as well as from the left versus right orbital rim (horizontal EOG). Electrode impedance was maintained below 5 kΩ. The EEG data were continuously sampled at 500 Hz and amplified using a 0.1–40 Hz band-pass filter. Eye blinks were removed from the EEGs using liner regression procedure [26]. Trials with peak-to-peak deflection exceeding ± 80 µV were excluded from averaging.

### ERP analysis

EEG of two standard auditory stimuli were segmented and averaged separately for the five attention conditions. ERP waveforms were time-locked to the onset of each standard stimulus and the average epoch was 2200 ms or 3200 ms, both including a pre-stimuli baseline of 200 ms. Due to the specific experimental manipulation, there was no correct response for the pitch discrimination. Thus epochs were averaged irrespective of response. According to the grand-mean ERP topographies and relevant literatures [19–21,27], the following six electrode sites were chosen for statistical analysis: Fz, F1, F2, FCz, FC1, and FC2. The peak amplitudes of the P2 component were automatically detected within a given time window of 150-250 ms while the amplitudes of CNV were estimated using area amplitude based on the integral under the ERP waveforms between two zero crossing points on the time axis [28].

### Source-localization analysis

The sLORETA is a 3-D discrete linear solution for the EEG inverse problem [29], which has shown significant correspondence with the neuroimaging results within the same tasks [30–32]. It computes the standardized current density in each of 6,239 voxels at 5 mm spatial resolution of the digitized Montreal Neurological Institute (MNI) standard brain [33,34]. The sLORETA performs a voxel-wise randomization test (5,000 permutations) based on the statistical non-parametric mapping (SnPM) [35]. In order to identify the neural mechanisms underlying the attention modulation on timing, the sLORETA images were compared between T and P conditions.

### Statistics

Statistical analyses were performed on SPSS Statistics 20.0 (IBM, Somers, USA). Descriptive data were presented as mean ± standard deviation (SD) or median (25th-75th percentile), as appropriate. The significance level was set at 0.05.

Nonparametric statistical tests were used to analyze the behavioral measurements of response time (RT) and accuracy rates (ACC), because individual RT and ACC were not normally distributed (as assessed by using the Lilliefors test). One-way analysis of variance (Friedman’s ANOVA) and the associated post-hoc testing (Wilcoxon Signed-ranks test) were conducted on RT and ACC measurements among attention conditions (T, Tp, tp, tP for time discrimination task and Tp, tp, tP, P for pitch discrimination task). Post-hoc testing of significant main effects was conducted using Bonferroni correction.

For ERP analysis, three-way repeated-measures ANOVA was conducted, using attention condition (T, Tp, tp, tP, P), electrode site, and tone duration (1500 ms, 2500 ms) as three factors. Greenhouse-Geisser correction for ANOVA tests was used whenever appropriate. Post-hoc testing of significant main effects was conducted using Bonferroni method. Significant interactions were analyzed using simple effects models. Partial eta-squared (η_*p*_^2^) was reported to demonstrate the effect size in ANOVA tests, where 0.05 represents a small effect, 0.10 indicates a medium effect, and 0.20 represents a large effect [36]. For the sake of brevity, effects that did not reach significance have been omitted.

## Results

### Behavior

**Figure 2:**
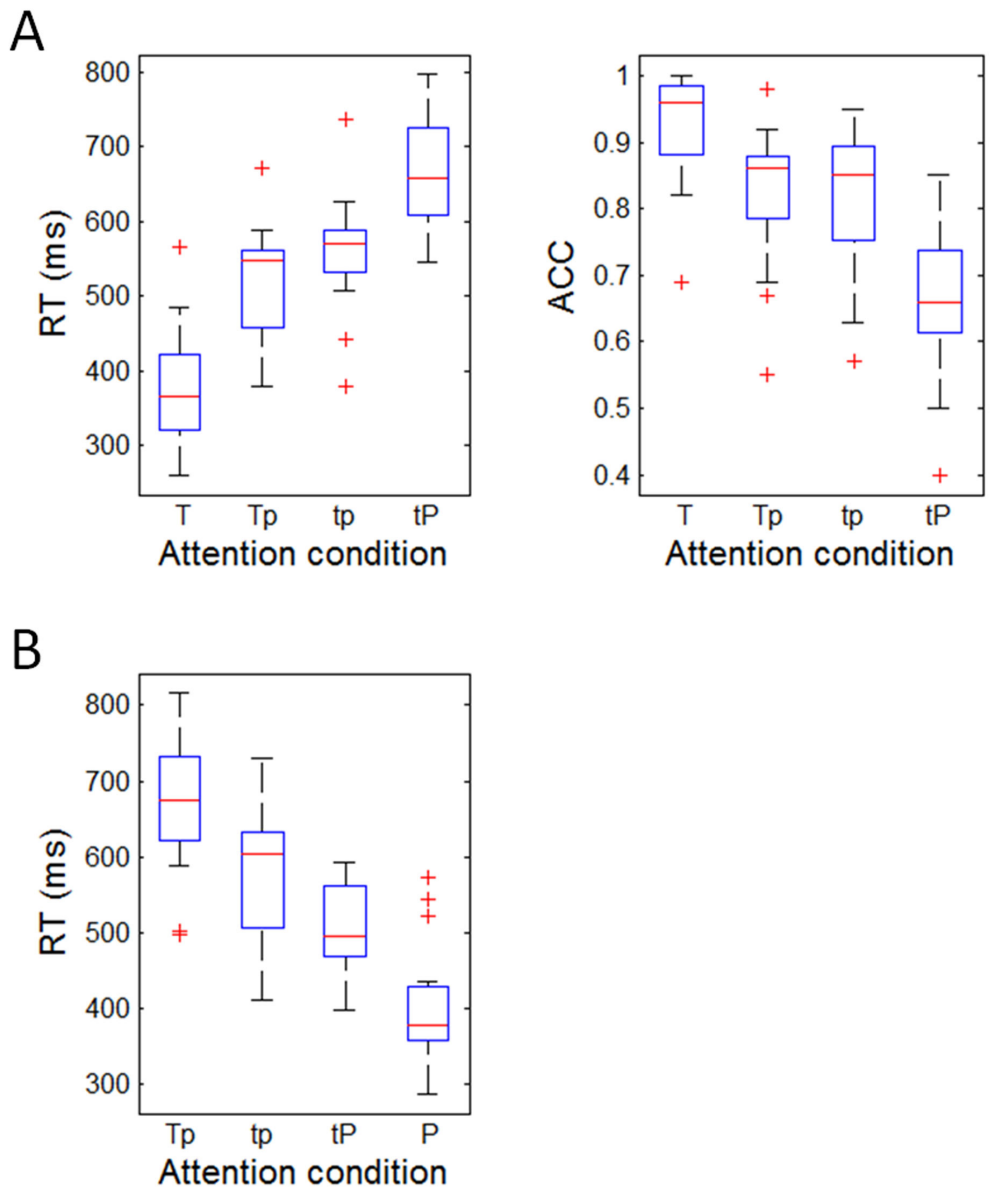
Behavioral results. A, the measurements of reaction time (RT) and accurate rate (ACC) in time discrimination task. B, the RT in pitch discrimination task. Shown are the boxplots of medians of RT and ACC in different attention modulation conditions. The three horizontal lines in every box reflect the lower quartile (25%), median (50%), and upper quartile (75%) of the statistics, respectively. The whisker is equal to one interquartile range of the statistics. The “+” marks represent outliers outside of the whisker.

The results in Figure 2 indicated that subjects allocated attention appropriately in the five attention conditions. Friedman’s ANOVA showed that with progressively decreasing attention to the time duration, participants responded with gradually slow of RTs in the time discrimination task (χ^2^(3,15) = 40.76, *p* < 0.001) (Figure 2A). Subjects responded the most quickly in the T condition (Median, 25th-75th percentile; 365 ms, 308-427 ms), less quickly in the Tp (547 ms, 451-562 ms) and tp conditions (569 ms, 523-590 ms), and the most slowly in the tP condition (658 ms, 604-739 ms). Post-hoc testing showed that all the pairwise comparisons were significant (Z = -2.44 to -3.41, *p* = 0.001-0.015). Meanwhile, with progressively increasing attention to pitch, subjects’ RT changed significantly in the pitch discrimination task (χ^2^(3,15) = 30.92, *p* < 0.001) (Figure 2B). Subjects responded the most quickly in the P condition (378 ms, 355-435 ms), less quickly in the tP (494 ms, 468–567 ms) and tp conditions (603 ms, 501-633 ms), and the most slowly in the Tp condition (674 ms, 621-739 ms). Post-hoc testing showed that all the pairwise comparisons were significant (Z = -2.04 to -3.41, *p* = 0.001-0.041).

Furthermore, the difference between global RTs of time (557 ms, 442-609 ms) and pitch discrimination tasks (526 ms, 450-632 ms) was not significant (Z = -0.04, *p* = 0.971), implying that the two tasks were matched for overall attention load, as was suggested by Coull et al. [7].

Similarly, increasing attention to time enhanced the ACC in time trials (χ^2^(3,15) = 26.05, *p* < 0.001) (Figure 2A). Specifically, subjects responded the most accurately in the T condition (96%, 88-99%), less accurately in the Tp (86%, 78-88%) and tp conditions (85%, 75-90%), and the least accurately in the tP condition (66%, 60-75%). Post-hoc testing showed that except the difference between Tp and tp condition (Z = -0.66, *p* = 0.509), all other pairwise comparisons were significant (Z = -2.70 to -3.41 *p* = 0.001-0.007). The ACC of pitch discrimination was not computed since there was no correct response in 80% pitch trials.

### ERP

#### *P2*

**Figure 3:**
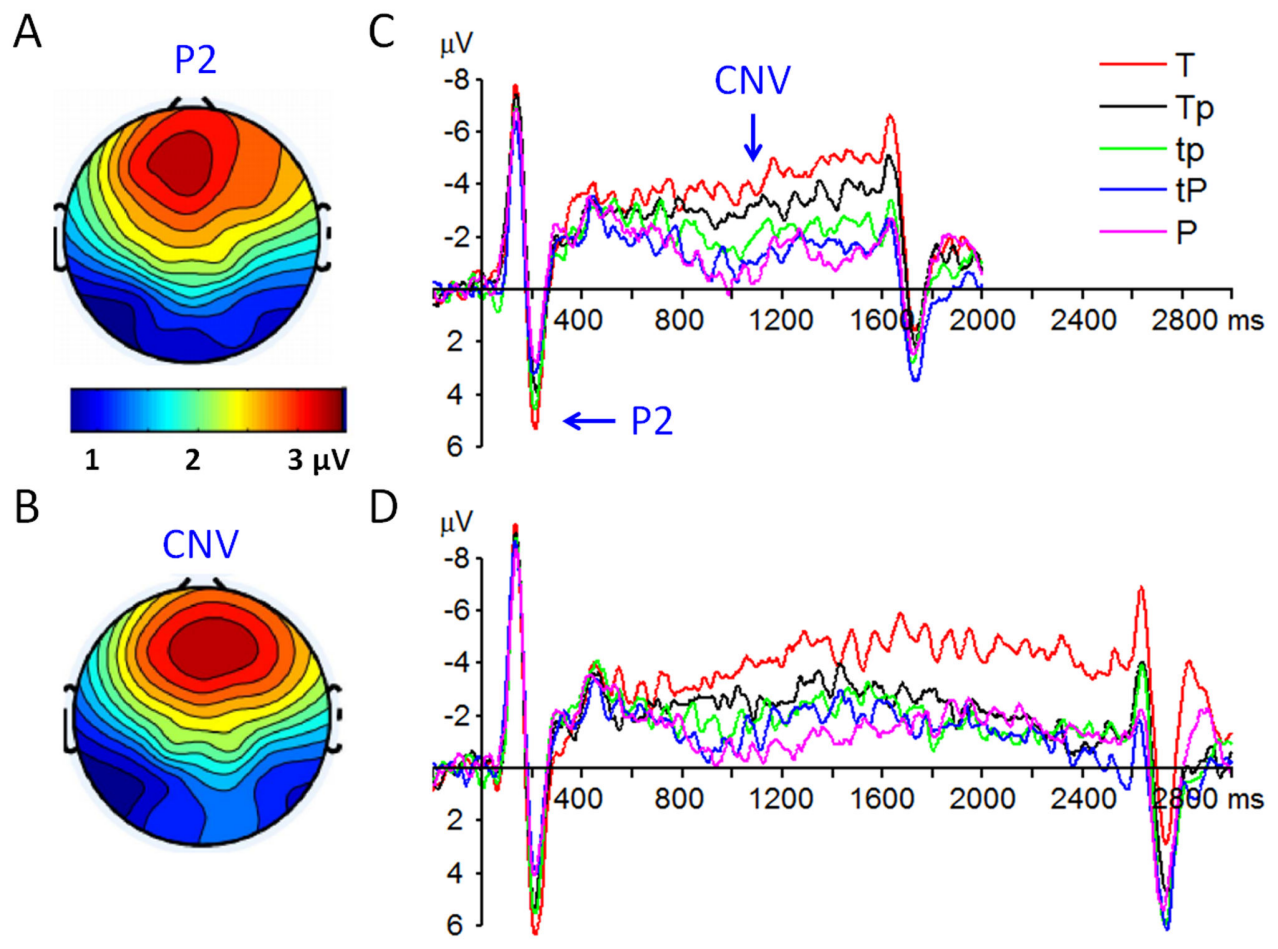
Grand-mean ERP waveforms and topographies of the P2 and CNV components. A, the P2 topography at 180-220 ms post-stimuli. B: the CNV topography at 800-1600 ms post-stimuli. C, the ERP waveforms (at FCz electrode site) in response to the 1.5-s tone. D, the ERP waveforms (at FCz electrode site) in response to the 2.5-s tone.

A repeated-measures 2 × 5 × 6 ANOVA was performed on the P2 amplitude with target duration, attention condition and electrode site as three within-subjects factors. The main effect of target duration was significant (F(1,14) = 7.19, *p* = 0.018, η_*p*_^2^ = 0.34), the P2 was larger in response to the 2.5-s tone (5.34 ± 3.67 µV) compared with the 1.5-s tone (4.27 ± 3.40 µV). The main effect of attention modulation was significant (F(4,56) = 4. 34, *p* = 0.004, η_*p*_^2^ = 0.24) (Figure 3E and F); the P2 amplitude was the largest in the T condition (5.95 ± 3.51 µV), smaller in the Tp (5.19 ± 3.74 µV), tp (5.05 ± 4.11 µV), and tP (4.05 ± 3.74 µV) conditions, and was the smallest in the P condition (3.79 ± 3.59 µV). Post-hoc testing showed that only the difference between T and P conditions was significant (*p* = 0.038).

#### *CNV*

**Figure 4:**
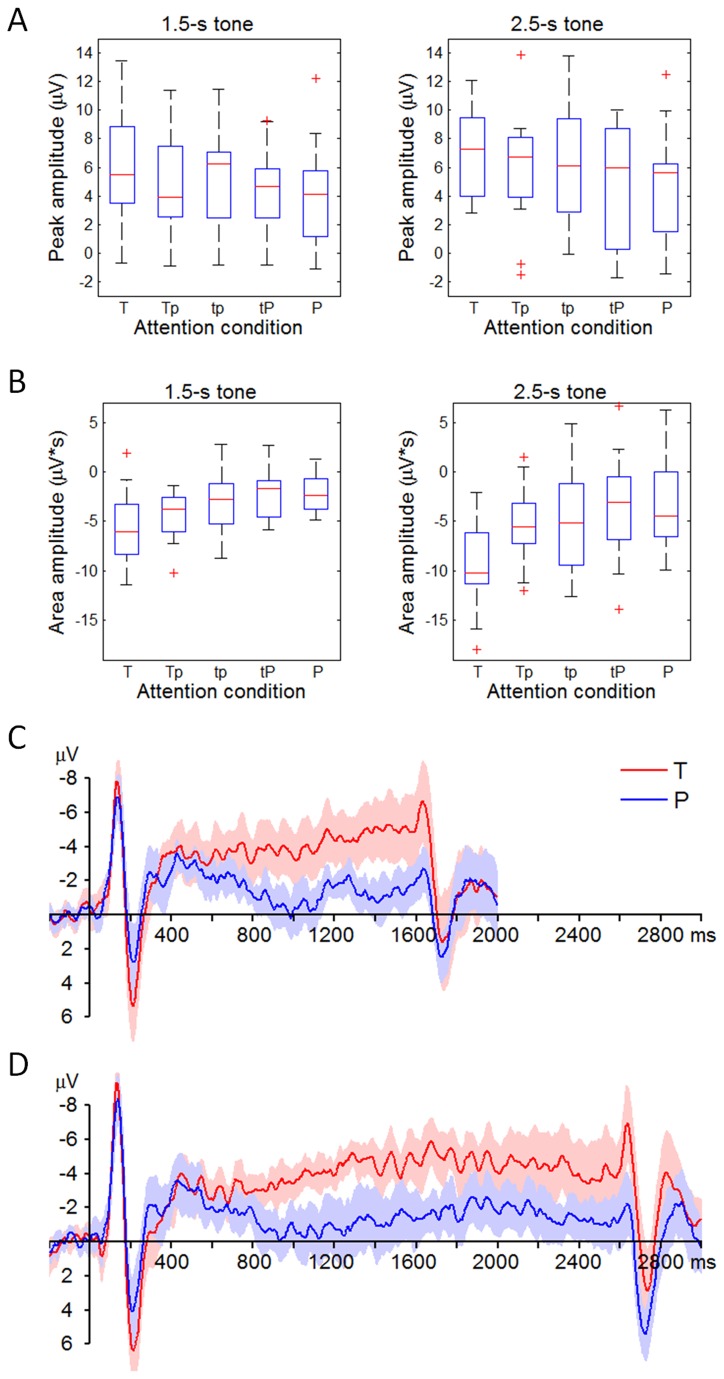
Distribution of ERP measurements in different attention conditions (data are collected at the electrode of FCz). A, the distribution of P2 amplitudes. The three horizontal lines in every box reflect the lower quartile (25%), median (50%), and upper quartile (75%) of the P2 amplitudes (refer to the legend of Figure 2 for more details). B, the distribution of CNV amplitudes. C, the average and 95% confidential interval (CI) of the ERPs in response to the 1.5-s tone. For the sake of brevity, only the ERPs in the T and the P conditions are displayed. D, the average and 95% CI of the ERPs in response to the 2.5-s tone.

A repeated-measures 2 × 5 × 6 ANOVA was performed on the CNV amplitude with target durations, attention condition and electrode site as the within-subjects factors. The main effect of target duration was significant (F(1,14) = 9.07, *p* = 0.009, η_*p*_^2^ = 0.39), the CNV was larger in response to the 2.5-s tone (6.20 ± 3.10 µV·s) compared with the 1.5-s tone (4.03 ± 1.91 µV·s). The main effect of attention modulation was significant (F(4,56) = 6.96, *p* < 0.001, η_*p*_^2^ = 0.33) (Figure 3E and F); the CNV amplitude was the largest in the T condition (8.34 ± 3.74 µV·s), smaller in the Tp (5.10 ± 2.95 µV·s), tp (4.53 ± 3.44 µV·s), and tP (3.99 ± 3.03 µV·s) conditions, and was the smallest in the P condition (3.61 ± 2.99 µV·s). Post-hoc testing showed that the T condition was significantly different with the other four attention conditions (*p* = 0.010-0.028). The P2 and CNV amplitudes in each attention condition were further plotted in Figure 4.

### Source localization

**Figure 5:**
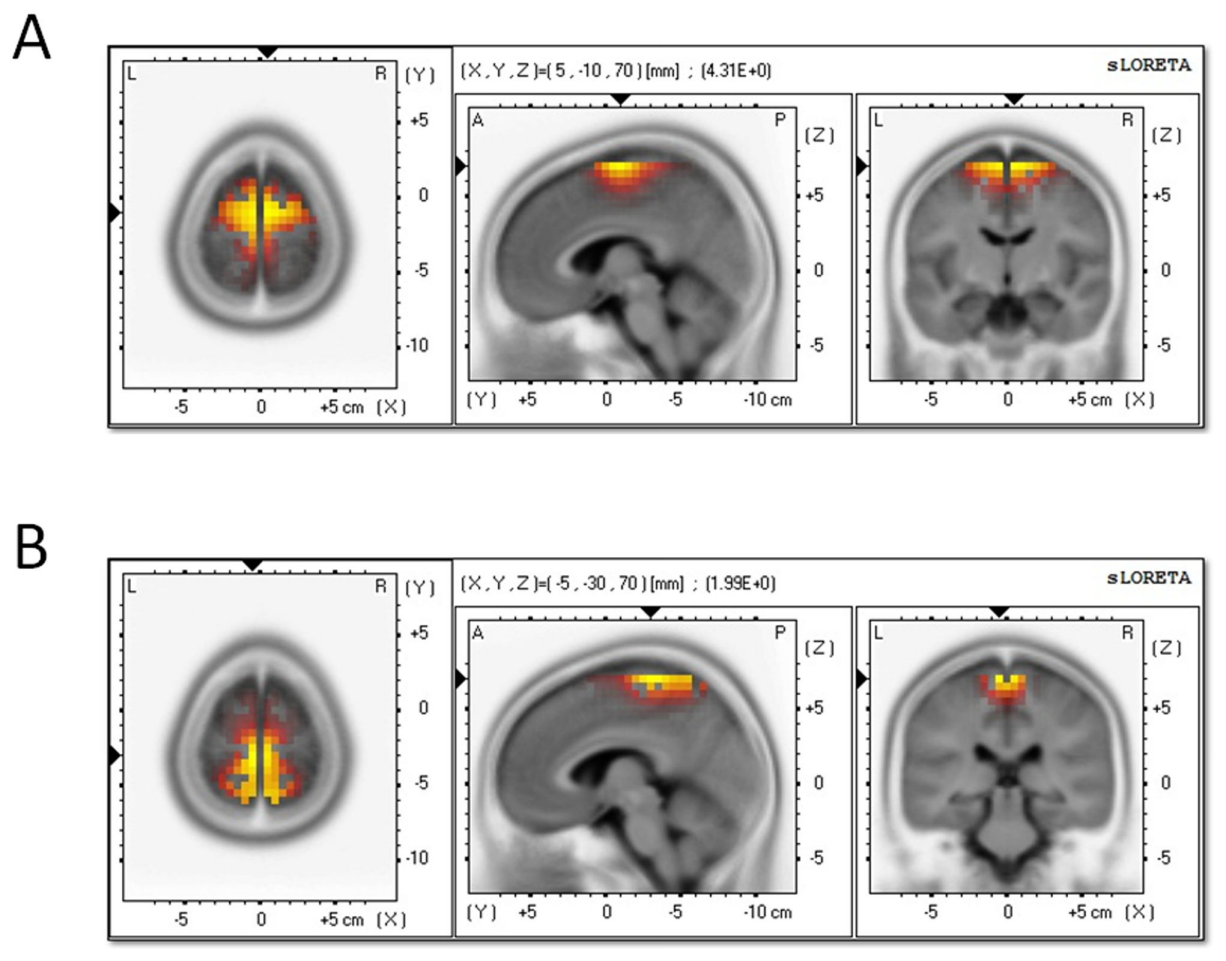
sLORETA images of the standardized current density maximum in the T vs. the P attention conditions. A, the results at the time interval of P2 (180-220 ms). B, the results at the time interval of CNV (800-1600 ms). The color scale is equal in all the maps, of which the strongest activations are indexed in yellow.

The voxel-based sLORETA images were compared between T and P conditions in order to identify the cortical regions responsible for timing. F values of the activated regions were listed in Table 1. As illustrated in Figure 5, the superior frontal gyrus (BA6; MNI coordinates: x = 5, y = -10, z = 70) was significantly activated at the time interval of the P2 component (Log-F-ratio = 4.31, p < 0.001) while the medial frontal gyrus (BA6; MNI coordinates: x = -5, y = 30, z = 70) was significantly activated at the time interval of the CNV component (Log-F-ratio = 1.96, p = 0.03).

**Table 1:** sLORETA results of the comparison between the T and P conditions in the P2 and CNV time intervals.

Anatomical region	MNI Coordinates	Log-F-ratio	Voxel number
P2 component:			581
BA 6	(5, -10, 70)	4.31^*^	
BA 6	(-9, -14, 60)	2.73^*^	
BA 6	(2, -9, 60)	2.66^*^	
BA 6	(0, -25, 55)	2.56^*^	
CNV component:			375
BA 6	(-5, 30, 70)	1.96^*^	
BA 6	(27, 5, 45)	1.88^*^	
BA 7	(10, -55, 55)	1.74^*^	

* *p* < 0.05. BA: Brodmann area. MNI: Montreal Neurological Institute coordinates. Voxel number indicates the exact number of activated voxels. Log-F-ratio, a log transformation of traditional F distribution; a larger Log-F-ratio represents a more significant activation in the brain. The Log-F-ratio at p 0.05 was 1.43 in current study.

## Discussion

Behavioral results showed that the attention was allocated appropriately in both tasks, suggesting that the attention modulation was effective and the experimental procedure was legitimate.

Two ERP components (P2 and CNV) were identified and analyzed in this study. The main effect of attention modulation was significant in P2 amplitudes. The P2 component was the largest in the T condition and the smallest in the P condition, indicating that the attention modulation on timing happened at an early stage of time perception, approximately at 200 ms after stimulus presentation. In general, the P2 is assumed to reflect the amount of cognitive resources allocated to perceptual processing of auditory features [18]. The current study further proved that the P2 can be considered as an early neural correlate of attention modulation on timing. However, the P2 sensitivity to attention modulation may be low, since the post-hoc testing showed that only the difference between the T and P conditions was significant.

The main effects of attention modulation and target duration were significant in the CNV amplitudes. The CNV was larger in response to the 2.5-s tone compared with the 1.5-s tone; the CNV amplitudes developed as a function of the attention allocated to timing. Statistical result indicated that the CNV was more sensitive to the attention effects on timing, as compared with the P2 component, thus can be considered as a more reliable index of attention modulation. In the current study, responses were required to be made with a delay of 1000 ms after the offset of the pure tone. Therefore, the decision-making phase was, to a large extent, separated from the pulse-accumulation phase. As a result, we suggested that the differentiated CNV amplitudes in various attention conditions were likely to be caused by the neural activity differences at the pulse-accumulation phase (not the decision-making phase). However, the present data only indicated that the CNV amplitude was sensitive to attention effects on timing; we could not decide between the decision-making hypothesis and the time estimation hypothesis. Further studies are needed for the exact nature of the relation between CNV and the underlying timing mechanisms.

In addition, the ERP source localization found that the attention to time vs. to pitch significantly activated the neural activity in superior (the P2 time interval) and medial frontal gyrus (the CNV time interval), both of which contained the cerebral region of SMA. It has been proved that the SMA usually relates to higher-order motor control processes in time perception, such as motor preparation and time sequencing [37]. However, evidence from recent neuroimaging studies suggested that the SMA not only relates to time production, but also is involved in the perception of temporal features of a stimulus [5,38–40]. These findings are is in line with our results. In the current study, it was highly unlike that the difference of SMA activation across different attention conditions be attributed to motor preparation, because the motor preparation was carefully controlled as described above. Therefore, the data in this study suggested that the SMA is an essential brain structure for attention-dependent quantification of time perception [19,20,22]. However, considering the inaccurate results of ERP source localization, this finding must be interpreted with cautious.

## Conclusions

The present study examined the neural mechanisms of attention modulation on timing using ERP and sLORETA measurements in a dual-task paradigm. The results showed that the attention modulation on timing may happen at an early stage, approximately 200 ms after stimulus presentation. The P2 component at frontal area served as an early neural correlate of attention modulation on timing. Compared to P2, the CNV at fronto-central area was more sensitive to the attention effects, thus can be considered as a potential ERP index of attention modulation. In addition, our data indicated that the SMA may be one of the key cerebral regions for attention-dependent quantification of time perception, though further neuroimaging studies are required to verify this source localization result in this study. The current findings improve our understandings of timing mechanisms and lay the ground for the investigation of patients with time perception deficits, such as attention-deficit hyperactivity disorder and Parkinson’s disease [41,42].
